# Invasion characteristics and clinical significance of tumor-associated macrophages in gastrointestinal Krukenberg tumors

**DOI:** 10.3389/fonc.2023.1006183

**Published:** 2023-02-24

**Authors:** Zigao Huang, Baojia Li, Haiquan Qin, Xianwei Mo

**Affiliations:** Guangxi Clinical Research Center for Colorectal Cancer, Division of Colorectal & Anal Surgery, Guangxi Medical University Cancer Hospital, Nanning, China

**Keywords:** Krukenberg tumor, tumor-associated macrophage, prognosis, immunohistochemistry, gastrointestinal cancer

## Abstract

**Background:**

Tumor-associated macrophages (TAMs) have been used as potential drug targets in preclinical research and clinical trials of various cancers. However, their distribution in Krukenberg tumors (KTs) remains unclear. We investigated the expression and prognostic value of TAMs in patients with gastrointestinal cancer with KTs.

**Methods:**

The infiltration of various types of TAMs was detected in surgical tissues of 35 patients with KTs using immunohistochemical staining. The level of infiltration of TAMs in tumor nests (TN), tumor stroma (TS), and invasive margin (IM) areas was evaluated. The Kaplan–Meier method and univariate/multivariate Cox regression risk models were used to analyze the relationship between the degree of TAMs invasion and overall survival (OS) and progression-free survival (PFS).

**Results:**

The distribution of TAMs exhibited spatial heterogeneity between TN, TS, and IM regions in primary tumor (PT) and KT tissues. TAMs infiltrated in the TN had greater prognostic value and were barely influenced by preoperative neoadjuvant therapy, despite similar grades of invasion in PT and KT tissues. Moreover, the number of CD68+ TAMs in TN of KT tissues was an independent risk factor affecting patient OS, whereas tumor resection scope might be an independent risk factor affecting patient PFS.

**Conclusions:**

In view of the close relationship between TAMs, the tumor microenvironment and patient prognosis, targeting TAMs combined with chemotherapy is expected to become a new approach for the treatment of patients with KTs.

## Introduction

Gastrointestinal (GI) cancer is one of the most common malignant tumors of the GI tract (also called digestive tract) worldwide. Diagnosis of a patient with advanced stage of the disease is often associated with the concomitant invasion or metastasis of the tumor to other organs, such as liver or lung (1, 2). Krukenberg tumors (KTs), which were first reported by German pathologist Friedrich Krukenberg in 1896, were considered an extremely rare type of metastatic tumors that colonized the ovaries of women with extra-ovarian malignancies (3). KTs usually affect both ovaries (bilateral) (4), accounting for approximately 1%–20% of all ovarian malignancies (5). In 1973, the World Health Organization updated the pathological diagnostic criteria for KTs to include: ovarian interstitial involvement, signed-ring cell carcinoma rich in mucous secretion, and sarcomatoid hyperplasia of ovarian interstitial (6). According to previous studies, the main source of KTs was gastric cancer (GC), followed by colorectal cancer (CRC), mucinous adenocarcinoma of the appendix, breast cancer, prostate cancer, and cervical cancer (7). In recent years, the incidence of CRC with KTs has been suggested to have gradually increased, even surpassing that of GC with KTs (8).

The tumor microenvironment is well known to include not only the tumor parenchyma, but also interstitial components surrounding tumor cells, such as immune cells, stromal cells, and fibroblasts, as well as the nutritional-related neovascularization of the tumor and a variety of specific biological factors (9). Recent studies have indicated that tumor stromal components can secrete various cytokines, as well as growth, tumor necrosis, and angiogenic factors through a variety of specific mechanisms, significantly affecting the biological behaviors of tumor parenchymal cells such as proliferation, migration, and invasion (10). Tumor parenchyma and tumor stroma (TS), which together constitute the niche of tumor cells, can both promote and restrict each other (11). In recent years, the interstitial composition of tumors has become an important research target for tumor therapy and intervention, broadening the prospects of anticancer research (12).

Tumor immune microenvironment (TIME) is an important component of the tumor microenvironment, with tumor-associated macrophages (TAMs) being the most common immune cells in TIME (13). Recent studies have indicated that macrophages not only provide vital innate immune defense and tissue homeostasis repair (14), but also directly or indirectly mediate tumor progression through the autocrine or paracrine secretion of exosomes and various cytokines (15), as well as through the induced radiation and chemotherapy resistance and immune tolerance of tumor cells (16). TAMs are thought to initially exist as the undifferentiated M0 type, and following exposure to certain stimuli are polarized to either of the two common subtypes, M1 and M2. The main phenotypes of the M1 type are iNOS+, CD11c+, and CD86+, which are activated by the classical pathway for killing tumor cells. The M2 type mainly includes CD163+, CD206+, and other phenotypes, which are activated by alternate pathways and play a role in promoting tumor growth. Importantly, M1 and M2 can be converted to each other under certain circumstances, that is, M2 is polarized to M1, and vice versa (14). Therefore, due to the unique effector functions of TAMs and their close relationship with malignant tumors, scientists have now genetically modified human macrophages and developed TAMs as a target for clinical antitumor therapy (17). In addition, high-density invasion of TAMs was positively associated with metastasis and reduced survival of most malignancies (18). Currently, studies on the prognostic significance of TAMs in patients with CRC remain controversial (19–21). These contradictory results might be attributed to different tumor types, different polarization patterns, and different distribution characteristics of TAMs in the tumor microenvironment (22).

In view of the close relationship between TAMs and malignant tumors, it is necessary to characterize the distribution characteristics of TAMs in tumors. TAMs have been reported in patients with CRC complicated with distant organ metastasis, including CRC complicated with liver (23, 24) and peritoneal (25) metastases. To the best of our knowledge, few studies have explored the correlation of the infiltration characteristics of TAMs in patients with CRC or GC combined with KTs. Hence, we retrospectively collected tissue samples from patients with GI cancer with KTs, and systematically detected the expression characteristics of specific TAM markers using immunohistochemical staining (CD68 marks pan-macrophages, CD11c marks M1 macrophages, whereas CD163 marks M2 macrophages). Kaplan–Meier and Cox regression analyses were conducted to explore the prognostic value of TAMs and reveal their potential role in the bioactive behavior of tumors, so as to provide insights for the treatment of KTs.

## Materials and methods

### Cohort design and participants

The study cohort included 35 patients with GI cancer with KTs who underwent mass resection in the Gastrointestinal Surgery Department of Guangxi Medical University Cancer Hospital from June 2014 to June 2020.

Inclusion criteria included (1): the primary lesion was located in the stomach or colorectum and was diagnosed as KTs following differentiation from primary malignant or benign ovarian tumor, as revealed by pathological examination and (2) clinical data were complete, pathological wax blocks were well-preserved, and long-term follow-up data were obtained.

Exclusion criteria included (1): other tumors or intestinal diseases (2); primary foci originating from other sites, such as breast, appendix, and cervix; and (3) patients who simply received medical treatment, out-of-hospital surgery, or just received pathological consultation in our hospital.

### Information collection and follow-up

Clinical information of patients, including age, body mass index, TNM stage, tumor location, pathological differentiation, lymph node metastasis, nerve invasion, vascular invasion, RAS status, and BRAF status, was retrospectively collected from medical records. Follow-up was carried out *via* telephone or by returning to the hospital for examination. The last follow-up was on July 12, 2021. Any metastasis with an interval of more than 3 months between the diagnosis of primary tumor (PT) and ovarian metastasis was defined as metachronous; otherwise, it was considered synchronous metastasis. The time from patients receiving first-line antitumor treatment to death from any cause was overall survival (OS), whereas the time of tumor progression, death from any cause, or time to receiving second-line treatment was progression-free survival (PFS). The Ethics Committee of Guangxi Medical University Cancer Hospital approved our study (LW2021078).

### Immunohistochemistry and staining evaluation

Paraffin specimens, including PTs, KTs, and contralateral “normal ovary” tissues that underwent prophylactic excision, were collected from the Pathology Department of Guangxi Medical University Cancer Hospital. After sampling, dehydration, embedding, and 4-μm thick sectioning, blank slides were made. Sections stained with hematoxylin and eosin (HE) were evaluated by an experienced pathologist.

Immunohistochemistry was performed using the ready-to-use fast immunohistochemistry MaxVisionTM2 assay kit (KIT-5920, MXB Biotechnologles, China). Specimens were first dewaxed, dehydrated, and repaired in a microwave for 15 min using EDTA (pH = 9.0). Each tissue specimen was then incubated with the following antibodies at 4°C overnight: CDX-2 (clone EPR2764Y, diluted 1:200, Abcam, USA), CD68 (clone BP6036, diluted 1:400, Biolynx, China), CD163 (clone BX50058, diluted 1:50, Biolynx), and CD11c (clone 2F1C10, diluted 1:4500, Proteintech, USA). DAB staining was performed by incubating at room temperature for 30 min using the secondary antibody contained in the kit.

**Figure 1:**
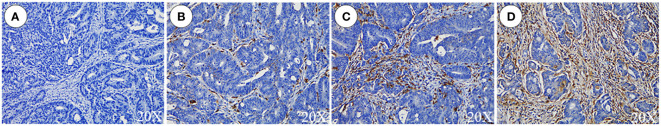
Grade scoring of CD68+ TAMs in tumor stroma of primary tumors. **(A)** None/sporadic. **(B)** Moderate. **(C)** Abundant. **(D)** Highly Abundant.

Cells with yellowish brown or brownish yellow granules in the nucleus or cytoplasm were positive cells. First, sections were evaluated as a whole under low magnification field of vision and the areas with the highest positive TAM density were selected for detailed observation. Next, the tumor nests (TN), tumor stroma (TS), and invasive margin (IM) were quantified under high magnification according to the evaluation method by Gill et al. (26). Scoring was performed as follows: none/sporadic = 1; moderate = 2; abundant = 3; highly abundant = 4, from which a total score was obtained. The different grade scoring standards of CD68+ TAMs in TS and PT are shown in **Figure 1**. All pathological sections were analyzed and interpreted by two senior pathologists in a double-blinded manner.

### Statistical analysis

Statistical analysis was conducted using the SPSS 26.0 software. Obtained data were tested for normal distribution using the Shapiro–Wilk test; those that met the normal distribution were expressed as the mean ± standard deviation, while differences between groups were evaluated using the unpaired two-sided *t*-test. Data that did not meet the normal distribution were expressed as the median (interquartile spacing) (M [P25-P75]), and differences between groups were evaluated using the Wilcoxon rank sum test. The chi-squared test or Fisher’s exact probability method was used for count data, whereas the Kruskal–Wallis or Wilcoxon rank sum test was used for continuous data. Hazard ratios (HR) and 95% confidence intervals (CI) were calculated in analyses using the univariate/multivariate Cox proportional risk regression model, with only the factors with P < 0.1 in univariate analysis being included in multivariate analysis. In addition, this study used an online analysis platform (https://www.xiantao.love/products), which is based on the R version 3.6.3 to analyze and visualize prognosis. The “Survival 3.2-10” and “SurvMiner 0.4.9” packages based on Kaplan–Meier analysis and log-rank test were used for statistical analysis and visualization of survival data, respectively. All performed tests were bilateral. P < 0.05 was considered statistically significant.

## Results

### Clinical characteristics

**Figure 2:**
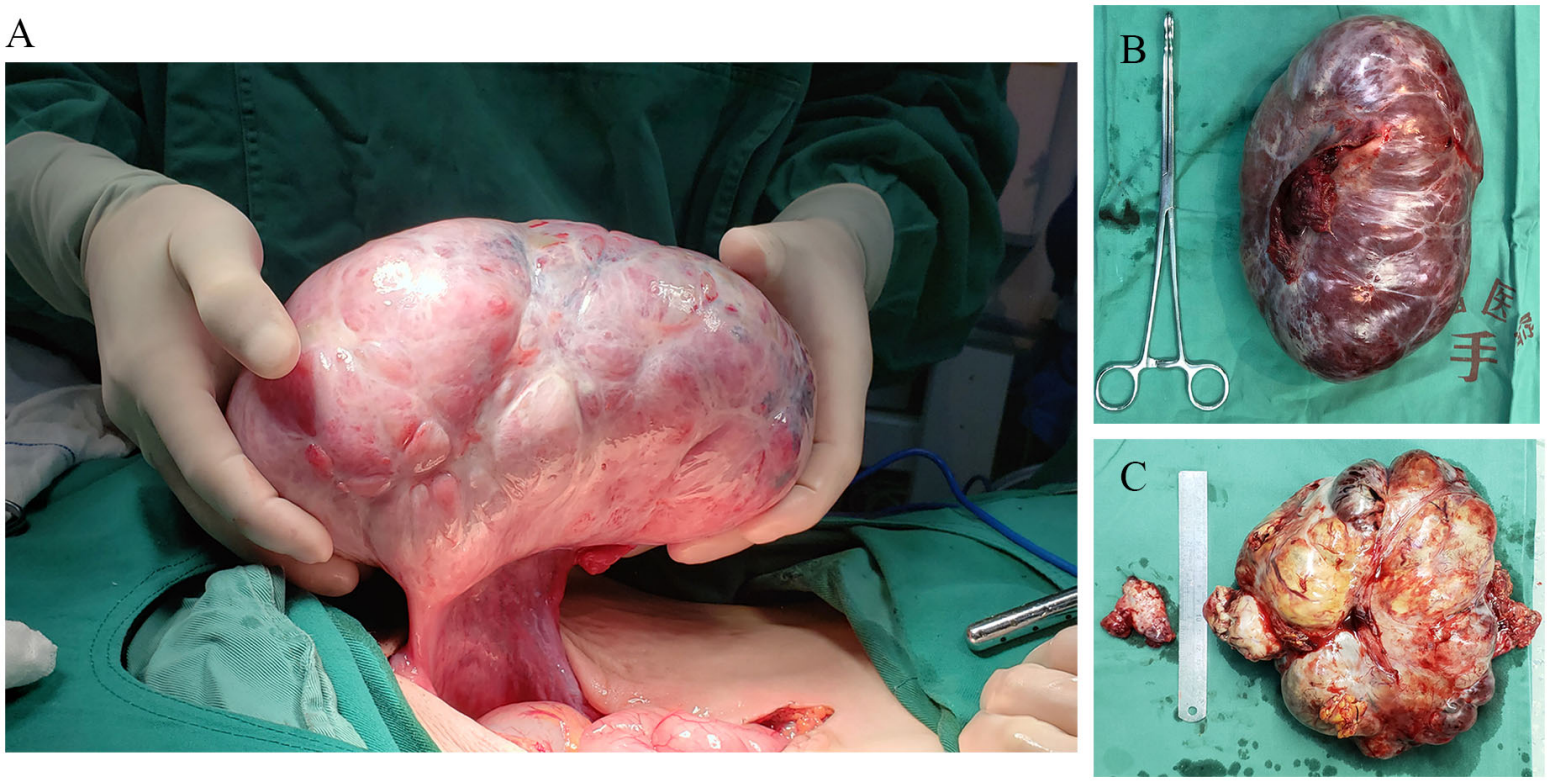
Representative samples of ovarian tissues from patients undergoing ovariectomy. **(A, B)** Patients were subjected to palliative resection of ovarian tumor alone. The ovarian tumor was large and kidney-shaped, with a complete capsule and smooth appearance without mass deposition. **(C)** Bilateral gross ovarian specimens from patients subjected to metastatic ovarian tumor resection and contralateral prophylactic oophorectomy. (left) Contralateral prophylactically resected ovarian tissue, (right) KT tissue.

We enrolled a total of 35 patients with KTs in our cohort, including 5 (14.3%) with GC and 30 with CRC (85.7%). The mean age of patients was 45.5 ± 12.4 years, ranging from 27 to 75 years. The diagnosis of KTs was often accompanied by lymphatic (n = 24, 68.6%), peritoneal (n = 18, 51.4%), and liver (n = 16, 45.7%) metastases. Oophorectomy was performed in 7 patients (31.4%), whereas 28 patients (68.6%) were subjected to complete or partial primary resection in addition to oophorectomy (**Figure 2**). It is worth noting that 19 patients (53.6%) received neoadjuvant therapy before surgery, whereas 33 (94.3%) received adjuvant therapy after surgery. Postoperative chemotherapy regimens mainly included oxaliplatin+capecitabine (FOLFOX6/mFOLFOX6) with or without bevacizumab/cetuximab, oxaliplatin+tegafur (SOX), and others. Clinical data are displayed in **Table 1**.

**Table 1:** Summary of patients’ characteristics.

Characteristics		No. of participant (%, n=35)	
Age	≥50	14	40.0%
	<50	21	60.0%
Menopausal status	Yes	17	48.6%
	No	18	51.4%
Primary tumor location	GC	5	14.3%
	colon cancer	13	37.1%
	sigmoid colon cancer	8	22.9%
	rectal cancer	9	25.7%
T stage	T2	1	2.9%
	T3	5	14.3%
	T4	29	82.9%
Unilateral/bilateral ovarian metastasis	Unilateral metastases	16	45.7%
	Bilateral metastases	19	54.3%
MMR status	pMMR	34	97.1%
	dMMR	1	2.9%
RAS status	Mutated	5	14.3%
	Wild type	11	31.4%
	NA	19	54.3%
lymphatic metastases	Yes	24	68.6%
	No	11	31.4%
Peritoneal metastases	Yes	18	51.4%
	No	17	48.6%
Liver metastases	Yes	16	45.7%
	No	19	54.3%
BRAF (V600E) status	Mutated	0	0
	Wild type	18	51.4%
	NA	17	48.6%
PI3K status	Mutated	0	0
	Wild type	13	37.1%
	NA	22	62.9%
Neoadjuvant therapy	Yes	19	54.3%
	No	16	45.7%
Adjuvant therapy	Oxaliplatin+capecitabine	14	40.0%
	Oxaliplatin+capecitabine+targeted drugs	10	28.6%
	Others	11	31.4%

MMR, mismatch repair; PI3K, phosphatidylinositol 3 kinase. NA, no applicable.

### Infiltration characteristics of TAMs in tissues

We eliminated five foci of KTs because we did not detect any cancer cells under the microscope and could not evaluate five foci of KTs for IM and total score due to lack of IM. Overall, we determined the abundance of TAMs in 28/28 PTs (100%) and 39/44 KTs (88.6%), whereas the abundance of TAMs in IM was reviewed in only 34/44 KTs (77.3%).

**Figure 3:**
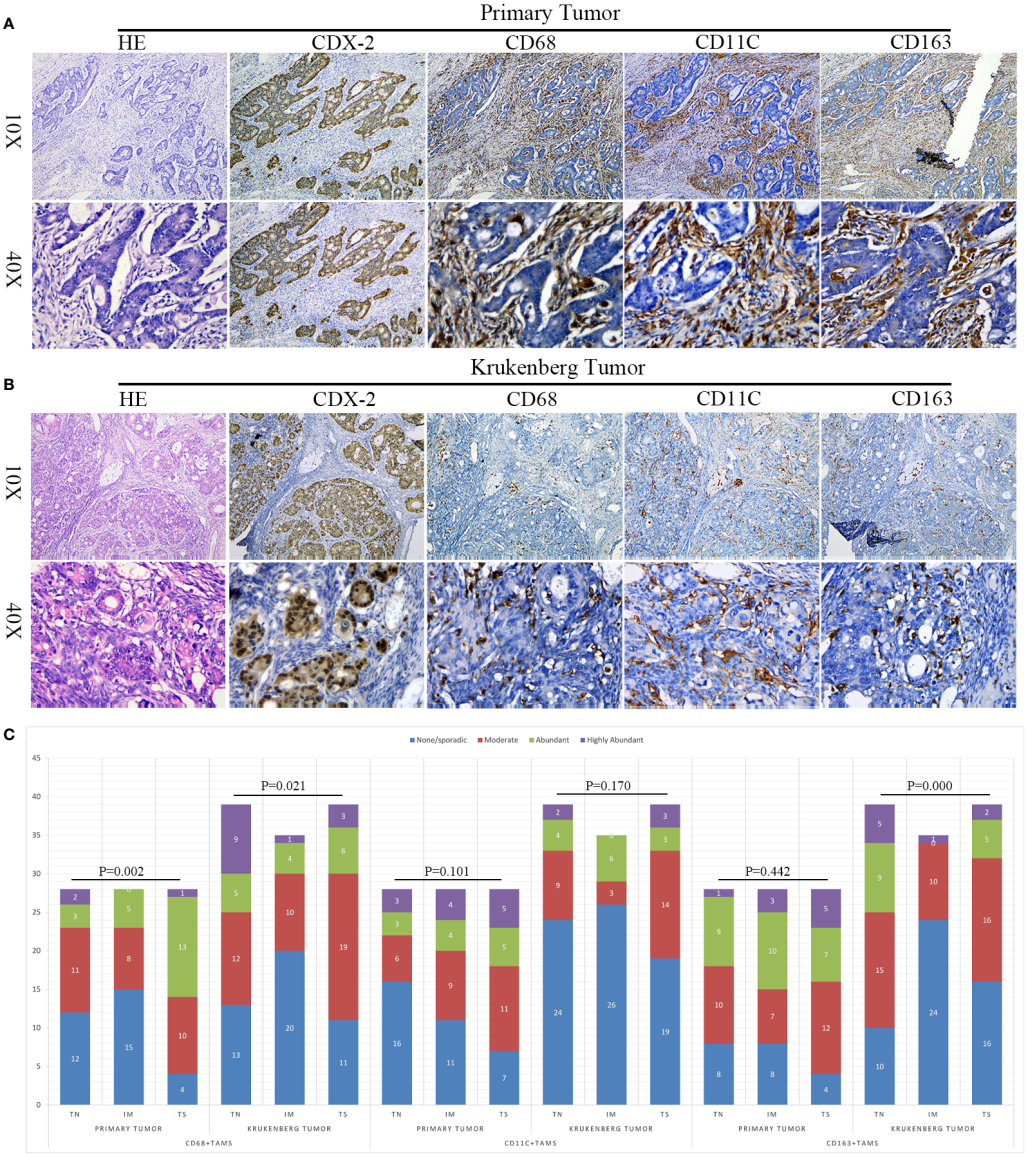
Expression and differential distribution of TAMs in primary tumors and KT tissues. **(A, B)** Representative images of HE, CDX-2, CD68, CD11c, and CD163 staining in primary tumor and KT tissues. **(C)** Spatial distribution characteristics of TAMs in primary tumors and KT tissues. The Y-axis denotes the number of cases.

Immunohistochemical staining indicated the expression of CD68, CD11c, and CD163 proteins in the nuclei and cytoplasms of TAMs, as demonstrated in **Figures 3A, B**. We found that TAMs exhibited different degrees of infiltration distribution in the PT and KT tissues. They were mainly distributed in the TS region, showing a scattered or fused patchy distribution, and occasionally some of them were fused with cancer cells. Morphologically, CD68+ and CD163+ TAMs generally showed a spindle or star shape, whereas CD11c+ TAMs showed a round or flat shape.

To analyze the distribution of various types of TAMs in TN, TS, and IM regions, we conducted a differential analysis. In PT tissues, we observed that the invasion of CD68+ TAMs was significantly different among TN, TS, and IM regions (P = 0.002), indicating spatial heterogeneity distribution. However, we did not detect any significant difference in the invasion of CD11c+ and CD163+ TAMs among this regions (P = 0.101 and P = 0.442, respectively). In KT tissues, CD68+ and CD163+ TAMs also presented spatial heterogeneity distribution in these three regions (P = 0.021, P = 0.000), whereas the differences in the distribution of CD11c+ TAMs among these three regions in KT tissues were not significant (P = 0.170), as demonstrated in **Figure 3C**.

**Figure 4:**
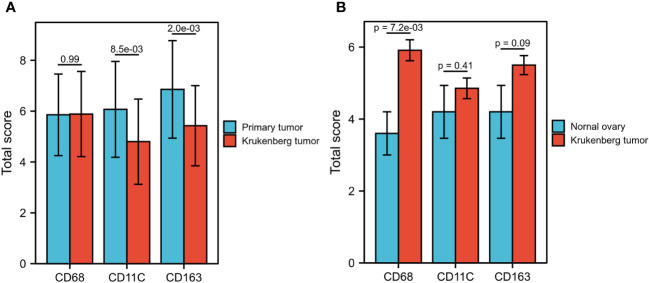
Comparison of CD68+, CD11c+, and CD163+ TAM scores between primary tumors, KT tissues, and prophylactically resected ovarian tissues. **(A)** The total score of CD11c+ and CD163+ TAMs in primary tumors was significantly higher than that in KT tissues (P = 8.5E-03, P = 2.0E-03), whereas no significant difference was observed in the total score of CD68+ TAMs (P = 0.99). **(B)** The total score of CD68+ TAMs in KT tissues was significantly higher than that in prophylactically resected ovarian tissues (P = 7.02-03), whereas no significant difference was observed in the total score of CD11c+ and CD163+ TAMs (P = 0.41, P = 0.09).

Subsequently, we analyzed the differences in the invasion of TAMs between PT and KT tissues. In the TN, we did not detect any statistical difference in the invasion of CD68+, CD11c+, and CD163+ TAMs between PT and KT tissues (P = 0.150, P = 0.603, and P = 0.699, respectively). In the TS, the invasion of CD68+, CD11c+, and CD163+ TAMs in PT tissues was greater than that in KT tissues (P = 0.046, P = 0.025, and P = 0.006, respectively). In the IM, we noticed that CD11c+ and CD163+ TAMs showed greater infiltration in PT than KT tissues (P = 0.009 and P = 0.000, respectively), whereas no significant difference was observed in the infiltration of CD68+ TAMs (P = 0.757) (**Table 2**). We further found that the total score of CD11c+ and CD163+ TAMs in PT tissues was higher than that in KT tissues (P = 8.5e-03 and P = 2.0e-03, respectively), whereas no significant difference was observed in the total score of CD68+ TAMs between PT and KT tissues (P = 0.99) (**Figure 4A**). In addition, compared with prophylactically resected ovarian tissues, CD68+ TAMs exhibited a higher total density score in KT tissues (P = 7.2e-03), whereas there was no significant difference in the total density scores of CD11c+ and CD163+ TAMs between the two tissues (P = 0.41 and P = 0.09, respectively) (**Figure 4B**). Correlation analysis between the levels of expression of various types of TAMs and clinicopathological indicators in TN of PT and KT tissues is provided in **Supplementary Materials 1**, **2**.

**Table 2:** Comparison of TAMs infiltration abundance between primary tumors and KT tissues.

		PT (n=28)				KT (n=39)				Z	*P*
				1 score	2 score	3 score	4 score	1 score	2 score	3 score	4 score
CD68	TN	12	11	3	2	13	12	5	9	-1.439	0.150
	IM	15	8	5	0	20	10	4	1	-0.309	0.757
	TS	4	10	13	1	11	19	6	3	-1.993	0.046
CD11c	TN	16	6	3	3	24	9	4	2	-0.520	0.603
	IM	11	9	4	4	26	3	6	0	-2.628	0.009
	TS	7	11	5	5	19	14	3	3	-2.248	0.025
CD163	TN	8	10	9	1	10	15	9	5	-0.387	0.699
	IM	8	7	10	3	24	10	0	1	-3.799	0.000
	TS	4	12	7	5	16	16	5	2	-2.763	0.006

TAMs, Tumor-associated macrophages; N, Number of patient; KT, Krukenberg tumor; PT, Primary tumor.

**Figure 5:**
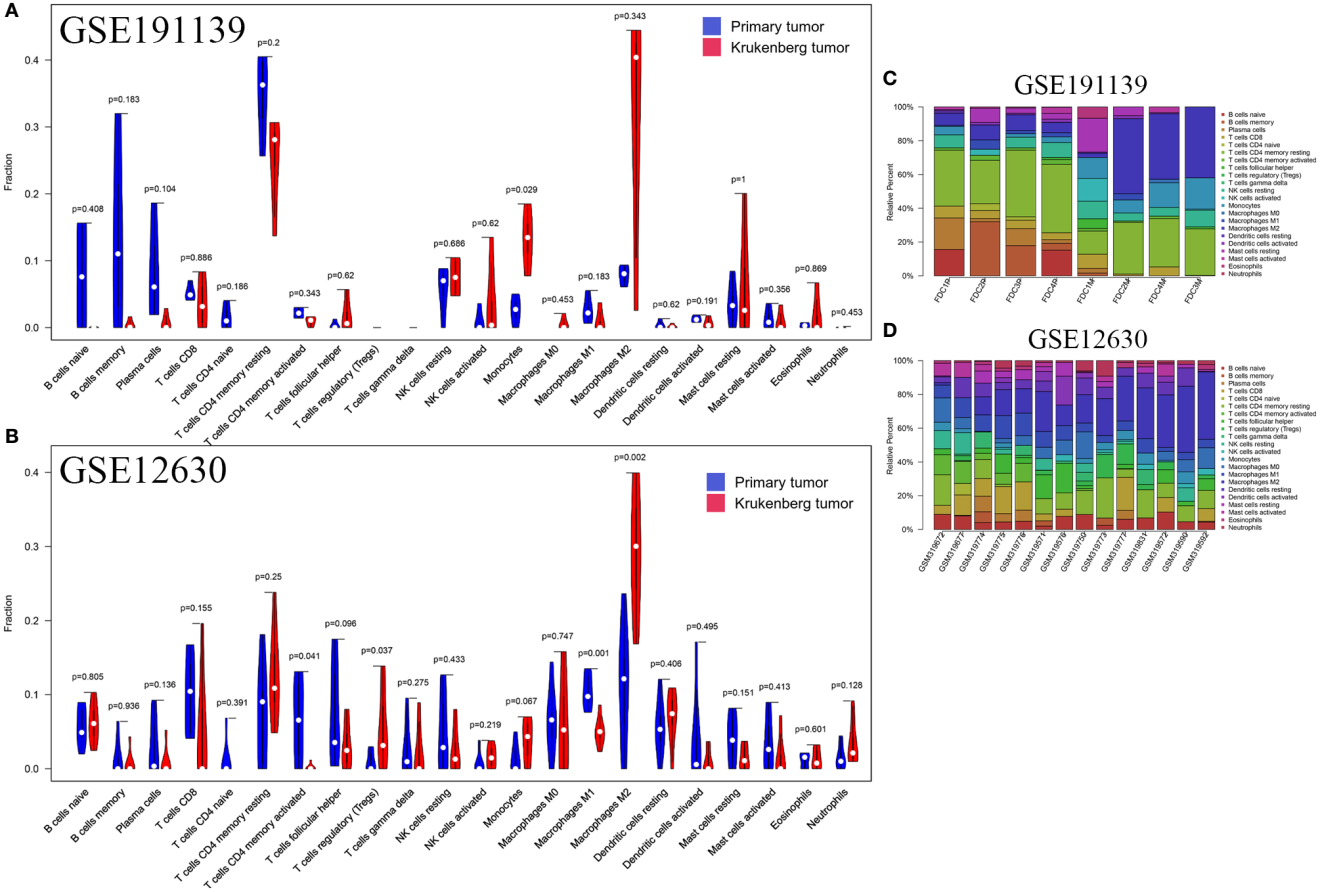
Relative proportion of 22 immune cells in primary tumors and KT tissues. **(A)** In the GSE191139 dataset, the numbers of M0 type macrophages (P = 0.453), M1 macrophages (P = 0.183), and M2 macrophages (P = 0.343) were similar in primary tumors and KT tissues. **(B)** In the GSE12630 dataset, M1 macrophages (P = 0.001) and M2 macrophages (P = 0.002) were more abundant in primary tumors than KT tissues, whereas no significant difference was observed in the number of M0 macrophages (P = 0.747). **(C, D)** Percentage of 22 types of immune cells in each sample.

To verify the difference in the number of immune cells between PT and KT tissues, we quantitatively analyzed 22 types of immune cells using published RNA sequencing (GSE191139) and gene expression profile chip (GSE12630) datasets. In the GSE191139 dataset, CIBERSORT results indicated that there were more scarce monocytes in PT than in KT tissues (P = 0.029), whereas no significant difference was observed in the numbers of memory B (P = 0.183) and helper T (P = 0.62) lymphocytes. We noticed that the density levels of TAMs (M0, M1, and M2) in PT and KT tissues were similar (P = 0.453, P = 0.183, and P = 0.343, respectively). In addition, in the GSE12630 dataset, PT tissues had more activated CD4+ memory T lymphocytes (P = 0.041) and fewer Tregs (P = 0.037) than KT tissues. Regarding macrophages, we found that compared with KT tissues, PT tissues had a higher number of M1 TAMs (P = 0.001) and lower number of M2 TAMs (P = 0.002); however, we did not detect any significant difference in the number of M0 TAMs between these tissues (P = 0.747) (**Figure 5**). Due to the small number of samples in these datasets and the inherent differences among different detection platforms, the reliability of these analyses although limited should have certain reference significance.

### Prognostic significance of infiltration level of TAMs in patients with KTs

In our study cohort, the median OS and PFS were 25 months (95% CI: 20.1–29.9 months) and 15 months (95% CI: 6.3–23.7 months), respectively. Based on the level of infiltration of TAMs, we divided patients into 1–2 (low expression) and 3–4 (high expression) groups. We used the Kaplan–Meier method to analyze the prognostic value of the level of infiltration of TAMs in PT and different regions of KT tissues for predicting OS and PFS in patients with KTs.

**Figure 6:**
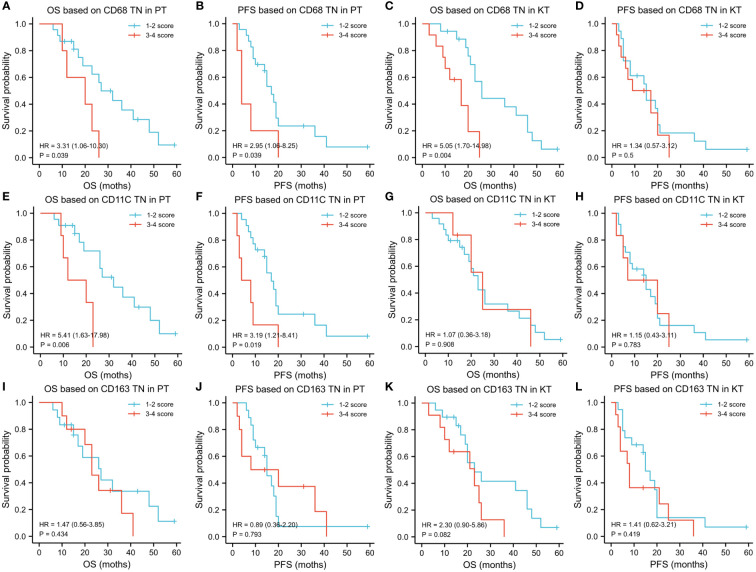
Influence of the level of infiltration of TAMs in tumor nest on prognosis of patients with KTs. **(A-L)** Kaplan–Meier analysis was used to explore the prognostic value of the infiltration of TAMs in the tumor nest region and different areas of primary tumors and KT tissues for OS and PFS. Regarding the tumor nest region of primary lesions, patients with KTs with high infiltration of CD68+ and CD11C+ TAMs had worse OS and PFS, whereas CD163+ TAMs had no significant effect on OS and PFS. Regarding the tumor nest region of KT tissues, the level of infiltration of CD68+ TAMs was related to the OS of patients but not to the PFS. No effect of the number of CD11c+ and CD163+ TAMs in the tumor nest of KTs on prognosis was observed.

We identified that patients with high invasion of CD68+ TAMs (OS: HR = 3.31 [1.06–10.30], P = 0.039; PFS: HR = 2.95 [1.06–8.25], P = 0.039) and CD11c+ TAMs (OS: HR = 5.41 [1.63–17.98], P = 0.006; PFS: HR = 3.19 [1.21–8.41], P = 0.019) had worse OS and PFS than patients with low infiltration in the TN of PT tissues. In contrast, we noticed that the level of infiltration of CD163+ TAMs had no significant correlation with OS and PFS of patients (OS: HR = 1.47 [0.56–3.85], P = 0.434; PFS: HR = 0.89 [0.36–2.20], P = 0.793). The level of infiltration of CD68+ TAMs in the TN of KT tissues was closely related to OS but not to PFS of patients (OS: HR = 5.05 [1.70–14.98], P = 0.004; PFS: HR = 1.34 [0.57–3.12], P = 0.50). Concomitantly, we did not detect any effect of the density of CD11c+ (OS: HR = 1.07 [0.36–3.18], P = 0.908; PFS: HR = 1.15 [0.43–3.11], P = 0.783) and CD163+ TAMs (OS: HR = 2.03 [0.90–5.86], P = 0.082; PFS: HR = 1.41 [0.62–3.21], P = 0.419) in TN of KT tissues on prognosis (**Figure 6**).

In addition, the level of infiltration of TAMs in TS and IM area had almost no effect on the OS and PFS of patients, as shown in **Supplementary Materials 3**, **4**. Therefore, we concluded that the infiltration of TAMs in TN has greater prognostic value.

### Univariate/multivariate Cox regression analyses

To further explore the prognostic value of multiple factors in patients with CRC or GC combined with KTs, we incorporated the above statistically significant indicators and clinical factors that might affect the OS and PFS of patients, such as age, resection scope, and CD68+ TAMs in TN of PT, into a Cox proportional risk regression model for univariate and multivariate analyses.

Regarding OS, univariate Cox proportional risk regression model suggested that the number of ovarian metastasis (HR = 0.423 [0.182–0.985], P = 0.046), PT location (HR = 4.053 [1.344–12.226], P = 0.013), CD68+ TAMs in TN of PT (HR = 0.318 [0.103–0.984], P = 0.047), CD68+ TAMs in TN of KT (HR = 0.200 [0.068–0.594], P = 0.004), CD11c+ TAMs in TN of PT (HR = 0.197 [0.060–0.647], P = 0.007), and CD11c+ TAMs in TS of PT (HR = 3.704 [1.149–11.940], P = 0.028) significantly affected the OS of patients with KTs. Of note, multivariate analysis suggested that the number of CD68+ TAMs in TN of KT tissues was an independent prognostic factor affecting the OS of patients (HR = 0.099 [0.010–0.941], P = 0.044) (**Table 3**).

**Table 3:** Univariate and multivariate analysis of the correlation of TAMs infiltration and clinicopathological factors with OS, PFS among patients with KT.

		OS				PFS				
Characteristics	N	Univariate analysis		Multivariate analysis		Univariate analysis				Multivariate analysis
		HR (95% CI)	P	HR (95% CI)	P	HR (95% CI)		P	HR (95% CI)	P
Age (<50 vs ≥50)	35	1.673 (0.708-3.956)	0.241			1.632 (0.735-3.621)		0.229		
Ovarian metastasis (One vs Two)	35	0.423 (0.182-0.985)	0.046	0.316 (0.084-1.193)	0.089	0.606 (0.285-1.289)		0.194		
Primary tumor location (GC vs CRC)	35	4.053 (1.344-12.226)	0.013	0.795 (0.097-6.545)	0.832	1.485 (0.555-3.971)		0.431		
Differentiation^a^	35	0.494 (0.218-1.119)	0.091			0.786 (0.367-1.682)		0.535		
Resection range (R0 vs Palliative resection)	35	1.693 (0.711-4.032)	0.235			1.981 (0.895-4.383)		0.092	3.283 (1.054-10.224)	0.040
Neoadjuvant therapy (Yes vs No)	35	0.897 (0.401-2.006)	0.791			0.931 (0.440-1.970)		0.851		
CD68 in TN of PT ^b^	28	0.318 (0.103-0.984)	0.047	1.287 (0.182-9.107)	0.801	0.355 (0.127-0.994)		0.049	1.182 (0.180-7.738)	0.862
CD68 in TN of PT ^b^	31	0.200 (0.068-0.594)	0.004	0.099 (0.010-0.941)	0.044	0.742 (0.318-1.729)		0.489		
CD11c in TN of PT ^b^	28	0.197 (0.060-0.647)	0.007	0.415 (0.066-2.625)	0.350	0.328 (0.124-0.865)		0.024	0.224 (0.036-1.393)	0.109
CD11c in TS of PT ^b^	28	3.704 (1.149-11.940)	0.028	2.205 (0.443-10.974)	0.334	1.317 (0.541-3.206)		0.544		
CD163 in TN of PT ^b^	28	0.691 (0.263-1.810)	0.451			1.103 (0.446-2.727)		0.832		

TAMs, Tumor-associated macrophages; OS, Overall survival; N, Number of patient; KT, Krukenberg tumor; GC, Gastric cancer; CRC, Colorectal cancer; ^a^High/medium differentiation vs Poor differentiated/mucous/signet ring cells; ^b^1-2 score vs 3-4 score.

Regarding PFS, univariate analysis suggested that the resection range (HR = 1.981 [0.895–4.383], P = 0.092), CD68+ TAMs (HR = 0.355 [0.127–0.994], P = 0.049), and CD11c+ TAMs (HR = 0.328 [0.124–0.865], P = 0.024) in the TN of PT were closely correlated with PFS. Multivariate analysis suggested that the resection range (HR = 3.283 [1.054–10.224], P = 0.040) was associated with PFS. In conclusion, we identified that the number of CD68+ TAMs in TN of KT tissues is an independent risk factor affecting OS in patients with CRC or GC combined with KTs, whereas tumor resection range might be an independent risk factor affecting PFS in patients (**Table 3**).

### Effect of preoperative neoadjuvant therapy on infiltration of TAMs and prognosis of patients

**Figure 7:**
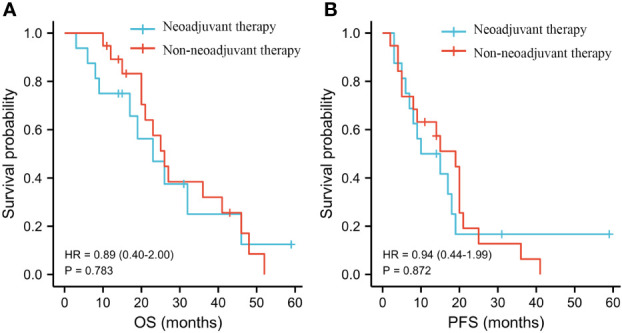
Influence of neoadjuvant therapy on the OS and PFS of patients. **(A)** OS. **(B)** PFS.

In our study cohort, 19/35 of patients (54.3%) received preoperative neoadjuvant therapy. Thus, in order to explore the influence of preoperative neoadjuvant therapy on the infiltration grade of TAMs and prognosis of patients, we conducted a differential analysis. We found that compared with the group without neoadjuvant therapy, the preoperative neoadjuvant therapy group had higher levels of infiltration of CD68+ TAMs in TN and TS of KT tissues (P = 0.041, P = 0.004). However, we did not detect any statistical significance in the comparison of the levels of infiltration of other types of TAMs in PT or KT tissues between the two groups (both P > 0.05) (**Supplementary Material 5**). Survival analysis showed that preoperative neoadjuvant therapy was not significantly associated with the OS (HR = 0.89 [0.40–2.00], P = 0.783) and PFS (HR = 0.94 [0.44–1.99], P = 0.872) of patients (**Figure 7**).

## Discussion

Recent scientific studies have confirmed that the level of invasion of TAMs in solid tumor tissues is an important indicator of the prognosis of patients with various cancers (27). Accordingly, therapeutic approaches targeting TAMs are expected to be part of the new strategies for future cancer treatment. In this study, using immunohistochemical staining, we detected the levels of expression of related markers of various types of TAMs in the tissues of patients with GI cancer complicated with KTs, revealing the distribution characteristics of TAMs in different polarization states in the tumor. In addition, our study also suggested that TAMs have a certain degree of spatial heterogeneity in PT and KT tissues of patients with GC or CRC combined with KTs, with TAMs in TN of PTs having higher invasion grade and better prognostic value than TAMs in KT tissues.

We initially evaluated the level of infiltration of TAMs in PT and KT tissues of patients with KTs. Our results indicated that TAMs had different degrees of density distribution in PT and KT tissues of patients and were mainly distributed in the tumor interstitial region but rarely in the ovarian tissues that had been prophylactically resected. Cancer is often considered as a disease closely related to chronic inflammatory processes. TAMs can be recruited into the tumor microenvironment after the colonization of tumor cells in metastatic organs to form an immune microenvironment and act as mediators of cancer-associated inflammation through the release of transcription factors (NF-κB, STAT3) that promote tumor growth. Angiogenic factor (VEGF-A), chemokines (CXCL8, CXCL12), and transforming growth factor-alpha (TGF-α) can also be produced to mediate immunosuppression (28, 29). Therefore, these mechanisms partly explain the increased infiltration of CD68+ TAMs in KT tissues than in prophylactically resected ovarian tissues. This was consistent with our previous finding that KTs with colonized cancer cells led to the accumulation of tumor-associated immune cells, whereas prophylactically resected ovarian tissues exhibited a small accumulation of tumor-associated immune cells. In view of previous studies, TAMs might be involved in the formation of the pretumor niche; therefore, prophylactic ovarian resection of the contralateral ovary is necessary for patients with KTs.

Further analysis demonstrated that the total score of M1 and M2 type TAMs, as well as their abundance in IM and TS in PT tissues, was significantly higher than those in KT tissues. In a recent study, Tai et al. (30) evaluated the expression of PD-L1 and levels of infiltration of T-cells in ovarian metastatic lesions and matched primary lesions of patients with KTs and found that the density levels of CD3+, CD8+, and FOXP3+ T– cells in PT tissues were significantly higher than those in KT tissues. These results supported the previous hypothesis that relevant immune cells in metastatic organs are recalled to the PT tissue after being “educated” in TIME (31), which might also be related to the low autoimmunogenicity and low susceptibility of ovarian tissues to aggregation of immune-related cells.

Our results suggested that the invasion of CD68+ TAMs in the proximal TN has better prognostic value than the invasion of CD68+ TAMs in the TS or IM regions. Previous studies have confirmed that high-density invasion of CD68+ TAMs in TN was associated not only with OS, PFS, and specific survival in patients with melanoma (32) but also with disease free survival in patients with endometrial cancer (33). Therefore, TAMs located in different regions of the same tumor lesion might have significantly different functional characteristics (34). In view of the above results, our study should have not only analyzed the degree of infiltration of TAMs but also emphasized the important influence of the location of the infiltration of TAMs on the prognosis of patients. However, the complex relationship between TAMs and immune cells, and between TAMs and tumor cells, as well as the related mechanisms in patients with KTs, remains to be further studied.

Donadon et al. (23) demonstrated that a larger area of TAMs in CRC liver metastases was closely associated with intratumoral single-cell diversity and poorer prognosis of patients. Combined with our observations, we concluded that compared with TAMs in the stroma and infiltrating margin of PT tissues, CD68+ TAMs in the TN tend to have more significant prognostic value. Moreover, analysis of their microstructure showed that CD68+ TAMs have larger cell morphology and mitotic features. Although the fusion with tumor cells might seem odd, it cannot be ruled out that the TAMs infiltrated in the TN are most frequently and closely cross-linked with tumor cells. Therefore, the relationship between the high infiltration of CD68+ TAMs in TN and the deterioration of survival outcomes of patients deserve our attention and further research.

Recent studies have indicated that M2 TAMs secrete various chemical factors, such as hypoxia-inducible factor–1α, vascular endothelial growth factor, and transforming growth factor–β, through a variety of molecular mechanisms to drive tumor vascular formation and EMT, and inhibit adaptive immune response, thus promoting the malignant behavior and function of tumor cells (35). In this study, we did not find any significant correlation between the numbers of CD163+ TAMs in different tissues and prognosis of patients with KTs. The negative results of this study were affected to some extent by the limited number of patients included; moreover, all patients included in this study had ovarian metastasis and were in the advanced stage of neoplastic disease, so TAMs in the immune microenvironment should be different from those in the early stage. Therefore, the failure of CD163+ TAMs to show significant prognostic value in patients in our cohort is not inconsistent with previous studies. Our study supported that high-density invasion of CD11c+ TAMs in the TN region of PT tissues was associated with poorer prognosis in patients with KTs, contrary to many previous studies that confirmed the tumor killing effect of M1 TAMs (36–38). However, this may also be similar to some previous literature reports that M1 TAMs may play a malignant pro-cancer role in some cancers (39–43). Thus, further experiments are needed to confirm this.

In general, PT tissues in patients with KTs have a greater infiltration of TAMs and stronger prognostic value than KT tissues. Therefore, in clinical practice, biopsy or palliative resection can be used to obtain PT tissue samples, followed by the evaluation of the expression of TAM-related markers in TN for predicting the PFS of patients, so as to better guide clinical treatment and TAM-targeting immunotherapy. However, for the comprehensive evaluation of the TN, TS and IM areas need to be excised to obtain larger specimens, which is undoubtedly impractical for unresectable patients. Concomitantly, our study examined whether the transformation of M2 into M1 is beneficial to survival. Although our results did not support the transformation of M2 into M1 or even the activation of TAMs, the strategy of targeting TAMs retained its theoretical feasibility in KTs.

Our study indicated that neoadjuvant therapy could significantly increase the invasion of CD68+ TAMs in the TN of PT tissues, whereas it had little or no effect on the invasion of TAMs in KT tissues. Although some tumor cells in the PT microenvironment are killed after neoadjuvant therapy, the released immunogenicity might activate the associated inflammatory response (44), potentially further exacerbating the recruitment of “educated” or “neoadjuvant therapy-survivor” TAMs in adjacent tumor cells. TAMs secrete many soluble molecules in the tumor microenvironment to protect tumor cells from drug attack (45–47), thus enabling them to achieve drug tolerance and immune escape. This TAM-associated “protection” might be one of the factors that preoperative neoadjuvant therapy does not fully benefit patients with KTs, and therefore early surgical resection should be the first choice of treatment. Despite the incomplete available information, the future development of strategies to overcome macrophage-associated immune tolerance might facilitate exploratory antitumor therapies.

However, our study had many shortcomings. As mentioned above, our study was a single-center, small-sample retrospective case study, which limited the reliability of our obtained results. Second, the analyses of this study were only performed at the theoretical level, lacking basic experiments to further verify the origin, occurrence, and development of KTs and drug resistance mechanisms of TAMs in depth. Moreover, these tumors were heterogeneous, and TAMs detected in small tissue specimens might not fully reflect the situation in the lesion site. The CD68, CD11c, and CD163 markers were selected to mark each type of TAMs based on previous studies. However, a single marker is not strictly a specific marker for a cell type, which may not be conducive to the localization and characterization of TAMs subtypes, and inevitably ignores the functional potential of other subsets (48). Therefore, a comprehensive and systematic characterization and localization of TAMs in different polarization states by multiple IHC or multicolor flow cytometry may be required in the future to more fully validate these promising results. Nonetheless, our study verified to some extent the infiltration distribution and prognostic value of various types of TAMs in the tissues of patients with KTs and suggested that TAM-targeting immunotherapy combined with other drugs might have beneficial effects in the treatment of patients with KTs.

## Conclusions

The mechanism of the interaction of TAMs with tumor cells, especially in TN, deserves further study. In view of the close relationship between TAMs, the tumor microenvironment, and patient prognosis, the therapeutic targeting of TAMs combined with chemotherapy is expected to become a new approach for the treatment of patients with GI cancer complicated with KTs.

## Data availability statement

The datasets presented in this study can be found in online repositories. The names of the repository/repositories and accession number(s) can be found in the article/**Supplementary Material**.

## Ethics statement

The studies involving human participants were reviewed and approved by Guangxi Medical University Cancer Hospital (LW2021078). The participants/patients provided their written informed consent to participate in the study.

## Author contributions

All authors contributed to the study conception and design. Concept and design: ZH. Data collection, analysis and interpretation, and manuscript writing: ZH, BL. Work supervision: XM, HQ. All authors contributed to the article and approved the submitted version.

## Supplementary material

Supplementary Material 1 Correlations between clinicopathological characteristics and infiltration of TAMs in TN of primary tumors.

Supplementary Material 2 Correlations between clinicopathological characteristics and infiltration of TAMs in TN of KT tissues.

Supplementary Material 3 Influence of the level of infiltration of TAMs in the tumor stroma region on prognosis of patients with KTs. (A-L) Kaplan–Meier method was used to analyze the prognostic value of the level of infiltration of TAMs in the tumor stroma region of primary lesions and KT tissues on the OS and PFS of patients. The infiltration density of CD11c+ TAMs was associated with the OS but not with PFS of patients, whereas other indicators had no significant correlation with patient outcomes.

Supplementary Material 4 Influence of the level of infiltration of TAMs in the invasive margin region on prognosis of patients with KTs. (A-J) Kaplan–Meier method was used to analyze the prognostic value of the level of infiltration of TAMs in the invasive margin region of primary lesions and KT tissues for OS and PFS. No association was observed between the level of infiltration of TAMs in the invasive margin region and patient outcomes.

Supplementary Material 5 Differences in infiltration of TAMs with or without neoadjuvant therapy.
