# Supplementary material for: Invasion characteristics and clinical significance of tumor-associated macrophages in gastrointestinal Krukenberg tumors
Source: Front Oncol. 2023 Feb 24;13:1006183. doi: 10.3389/fonc.2023.1006183 (PMC9999382; doi:10.3389/fonc.2023.1006183)
Supplement: Supplementary Material 1 — Correlations between clinicopathological characteristics and infiltration of TAMs in TN of primary tumors. [file Table_1.docx]

| Supplementary material 1. Correlations between characteristics and TAMs infiltration in TN of primary tumor | | | | | | | | | |
| --- | --- | --- | --- | --- | --- | --- | --- | --- | --- |
| Variable | CD68 | | | CD11c | | | CD163 | | |
|  | 1-2 score | 3-4 score | *P* | 1-2 score | 3-4 score | *P* | 1-2 score | 3-4 score | *P* |
| N | 23 | 5 |  | 22 | 6 |  | 18 | 10 |  |
| Menstrual status | |  | 1.000 |  |  | 0.648 |  |  | 0.695 |
| No | 11 | 3 |  | 12 | 2 |  | 10 | 4 |  |
| Yes | 12 | 2 |  | 10 | 4 |  | 8 | 6 |  |
| Pathological differentiation | |  | 0.050 |  |  | 0.008 |  |  | 0.035 |
| Moderate/ well | 16 | 1 |  | 16 | 1 |  | 14 | 3 |  |
| Poor | 3 | 3 |  | 2 | 4 |  | 2 | 4 |  |
| Signet-ring/mucinous | 4 | 1 |  | 4 | 1 |  | 2 | 3 |  |
| T stage |  |  | 1.000 |  |  | 1.000 |  |  | 1.000 |
| T1-3 | 5 | 1 |  | 5 | 1 |  | 4 | 2 |  |
| T4 | 18 | 4 |  | 17 | 5 |  | 14 | 8 |  |
| N stage |  |  | 1.000 |  |  | 1.000 |  |  | 1.000 |
| N- | 7 | 2 |  | 7 | 2 |  | 6 | 3 |  |
| N+ | 16 | 3 |  | 15 | 4 |  | 12 | 7 |  |
| KT size | |  | 1.000 |  |  | 0.655 |  |  | 1.000 |
| ＜10 cm | 12 | 3 |  | 11 | 4 |  | 10 | 5 |  |
| ≥10 cm | 11 | 2 |  | 11 | 2 |  | 8 | 5 |  |
| Tumor location | |  | 0.003 |  |  | 0.006 |  |  | 0.037 |
| CRC | 23 | 2 |  | 22 | 3 |  | 18 | 7 |  |
| GC | 0 | 3 |  | 0 | 3 |  | 0 | 3 |  |
| RAS status | |  | 1.000 |  |  | 1.000 |  |  | 1.000 |
| Mutant | 5 | 0 |  | 5 | 0 |  | 4 | 1 |  |
| Wild | 8 | 1 |  | 9 | 0 |  | 7 | 2 |  |
| Peritoneal metastasis | |  | 0.133 |  |  | 0.354 |  |  | 0.243 |
| No | 15 | 1 |  | 14 | 2 |  | 12 | 4 |  |
| Yes | 8 | 4 |  | 8 | 4 |  | 6 | 6 |  |
| Age* | 48.48 ± 12.84 | 37.4 ± 11.46 | 0.087 | 46.55 ± 13.34 | 46.33 ± 13.57 | 0.973 | 46.89 ± 11.8 | 45.8 ± 15.93 | 0.838 |
| BMI* | 21.8 ± 3.25 | 18.54 ± 2.61 | 0.046 | 21.69 ± 3.43 | 19.48 ± 2.59 | 0.156 | 21.64 ± 3.46 | 20.45 ± 3.17 | 0.376 |
| CEA (ng/ml) | 10.73 (3.6, 73.11) | 3.43 (0.81, 3.5) | 0.045 | 13.18 (4.02, 79.14) | 2.53 (1.01, 3.48) | 0.005 | 17.07 (3.43, 79.14) | 5.71 (2.64, 10.08) | 0.160 |
| Ki-67, % * | 70 (60, 80) | 70 (60, 75) | 0.843 | 70 (60, 80) | 60 (52.5, 67.5) | 0.138 | 70 (60, 80) | 60 (60, 73.75) | 0.438 |
| TAMs=[tumor-associated](javascript:;) [macrophage](javascript:;)s; N=number of patients; TN=tumor nets; BMI=body mass index; CEA=carcinoembryonic antigen; CA199=carbohydrate antigen199; Bold values indicate P < 0.05, *mean ±[standard deviation](https://www.baidu.com/link?url=wPn0Yf8nWQJWIBJo6hwPpyaiC5YjpaGG-1QqxOCzAYGnt2oN25J2gG5SFI5qLb8Fxbv5MPQCm75ioc4RYWXegRORI22tCkG4d_0tVIAhluYyrvvGjhypa2l51SglN3TS&wd=&eqid=81e61e35000303f000000006613e0596). | | | | | | | | | |
