# Supplementary material for: Invasion characteristics and clinical significance of tumor-associated macrophages in gastrointestinal Krukenberg tumors
Source: Front Oncol. 2023 Feb 24;13:1006183. doi: 10.3389/fonc.2023.1006183 (PMC9999382; doi:10.3389/fonc.2023.1006183)
Supplement: Supplementary Material 2 — Correlations between clinicopathological characteristics and infiltration of TAMs in TN of KT tissues. [file Table_2.docx]

| Supplementary material 2. Correlations between characteristics and TAMs infiltration in TN of KT | | | | | | | | | |  |
| --- | --- | --- | --- | --- | --- | --- | --- | --- | --- | --- |
| Variable | CD68 | | | CD11c | | | CD163 | | |  |
|  | 1-2 score | 3-4 score | *P* | 1-2 score | 3-4 score | *P* | 1-2 score | 3-4 score | *P* |  |
| N | 18 | 12 |  | 24 | 6 |  | 19 | 11 |  |  |
| Menstrual status | |  | 1.000 |  |  | 1.000 |  |  | 0.466 |  |
| No | 10 | 6 |  | 13 | 3 |  | 9 | 7 |  |  |
| Yes | 8 | 6 |  | 11 | 3 |  | 10 | 4 |  |  |
| Pathological differentiation | |  | 0.035 |  |  | 0.832 |  |  | 0.170 |  |
| Moderate/ well | 10 | 7 |  | 14 | 3 |  | 13 | 4 |  |  |
| Poor | 2 | 5 |  | 5 | 2 |  | 4 | 3 |  |  |
| Signet-ring/mucinous | 6 | 0 |  | 5 | 1 |  | 2 | 4 |  |  |
| T stage |  |  | 1.000 |  |  | 0.302 |  |  | 1.000 |  |
| T1-3 | 4 | 2 |  | 6 | 0 |  | 4 | 2 |  |  |
| T4 | 14 | 10 |  | 18 | 6 |  | 15 | 9 |  |  |
| N stage |  |  | 0.418 |  |  | 0.329 |  |  | 0.687 |  |
| N- | 4 | 5 |  | 6 | 3 |  | 5 | 4 |  |  |
| N+ | 14 | 7 |  | 18 | 3 |  | 14 | 7 |  |  |
| KT size | | | 1.000 |  |  | 0.651 |  |  | 0.450 |  |
| ＜10 cm | 9 | 6 |  | 13 | 2 |  | 8 | 7 |  |  |
| ≥10 cm | 9 | 6 |  | 11 | 4 |  | 11 | 4 |  |  |
| Tumor location | | | 1.000 |  |  | 1.000 |  |  | 0.047 |  |
| CRC | 15 | 10 |  | 20 | 5 |  | 18 | 7 |  |  |
| GC | 3 | 2 |  | 4 | 1 |  | 1 | 4 |  |  |
| RAS status | | | 0.217 |  |  | 0.203 |  |  | 0.266 |  |
| Mutant | 1 | 3 |  | 2 | 2 |  | 1 | 3 |  |  |
| Wild | 7 | 2 |  | 8 | 1 |  | 6 | 3 |  |  |
| Peritoneal metastasis | |  | 0.711 |  |  | 0.672 |  |  | 0.708 |  |
| No | 7 | 6 |  | 11 | 2 |  | 9 | 4 |  |  |
| Yes | 11 | 6 |  | 13 | 4 |  | 10 | 7 |  |  |
| Age* | 46.5 (33.25, 51.75) | 45 (37.5, 59.5) | 0.539 | 46 ± 12.86 | 43.67 ± 11.69 | 0.689 | 48 ± 11.12 | 41.27 ± 14.05 | 0.158 |  |
| BMI* | 20.71 ± 3.54 | 21.46 ± 3.09 | 0.556 | 21.25 ± 3.58 | 20.07 ± 2.01 | 0.448 | 21.51 ± 3.47 | 20.15 ± 3.02 | 0.292 |  |
| CEA (ng/ml) | 15.46 (8.1, 79.14) | 5.63 (3.1, 73.2) | 0.363 | 11.16 (4.16, 67.08) | 5.27 (3.45, 76.76) | 0.795 | 19.35 (3.73, 86.69) | 8.83 (5.23, 22.72) | 0.667 |  |
| Ki-67, % * | 70 (60, 80) | 65 (47.5, 71.25) | 0.217 | 70 (60, 80) | 70 (55, 70) | 0.935 | 70 (62.5, 80) | 60 (50, 67.5) | 0.072 |  |
| TAMs=[tumor-associated](javascript:;) [macrophage](javascript:;)s; N=number of patients; TN=tumor nets; BMI=body mass index; CEA=carcinoembryonic antigen; CA199=carbohydrate antigen199; Bold values indicate P < 0.05, *mean ±[standard deviation](https://www.baidu.com/link?url=wPn0Yf8nWQJWIBJo6hwPpyaiC5YjpaGG-1QqxOCzAYGnt2oN25J2gG5SFI5qLb8Fxbv5MPQCm75ioc4RYWXegRORI22tCkG4d_0tVIAhluYyrvvGjhypa2l51SglN3TS&wd=&eqid=81e61e35000303f000000006613e0596). | | | | | | | | | | |
