# Supplementary material for: Invasion characteristics and clinical significance of tumor-associated macrophages in gastrointestinal Krukenberg tumors
Source: Front Oncol. 2023 Feb 24;13:1006183. doi: 10.3389/fonc.2023.1006183 (PMC9999382; doi:10.3389/fonc.2023.1006183)
Supplement: Supplementary Material 3 — Influence of the level of infiltration of TAMs in the tumor stroma region on prognosis of patients with KTs. (A-L) Kaplan–Meier method was used to analyze the prognostic value of the level of infiltration of TAMs in the tumor stroma region of primary lesions and KT tissues on the OS and PFS of patients. The infiltration density of CD11c+ TAMs was associated with the OS but not with PFS of patients, whereas other indicators had no significant correlation with patient outcomes. [file Table_3.docx]

| Supplementary material 5. Differences in infiltration of TAMs with or without neoadjuvant therapy | | | | | | | | | | | | | | | | |
| --- | --- | --- | --- | --- | --- | --- | --- | --- | --- | --- | --- | --- | --- | --- | --- | --- |
| Group |  | *PT* | | | | | | |  | *KT* | | | | | | |
|  |  | Neoadjuvant therapy（n=14） | |  | Non-neoadjuvant therapy（n=14） | | X^2^ | *P* |  | Neoadjuvant therapy（n=17） | |  | Non-neoadjuvant therapy（n=13） | | X^2^ | *P* |
|  |  | 1-2分 | 3-4分 |  | 1-2分 | 3-4分 |  |  |  | 1-2分 | 3-4分 |  | 1-2分 | 3-4分 |  |  |
| CD68 | TN | 9 | 5 |  | 14 | 0 | - | 0.041 |  | 12 | 5 |  | 6 | 7 | 0.956 | 0.328 |
|  | IM | 11 | 3 |  | 12 | 2 | 0.000 | 1.000 |  | 13 | 4 |  | 11 | 1 | - | 0.370 |
|  | TS | 7 | 7 |  | 7 | 7 | 0.000 | 1.000 |  | 9 | 8 |  | 13 | 0 | - | 0.004 |
| CD11c | TN | 10 | 4 |  | 12 | 2 | 0.212 | 0.645 |  | 12 | 5 |  | 12 | 1 | - | 0.196 |
|  | IM | 8 | 6 |  | 12 | 2 | 1.575 | 0.209 |  | 14 | 3 |  | 11 | 1 | - | 0.622 |
|  | TS | 8 | 6 |  | 10 | 4 | 0.156 | 0.695 |  | 14 | 3 |  | 13 | 0 | - | 0.238 |
| CD163 | TN | 7 | 7 |  | 11 | 3 | 1.400 | 0.237 |  | 10 | 7 |  | 9 | 4 | 0.042 | 0.838 |
|  | IM | 9 | 5 |  | 6 | 8 | 0.570 | 0.449 |  | 16 | 1 |  | 12 | 0 | - | 1.000 |
|  | TS | 8 | 6 |  | 8 | 6 | 0.000 | 1.000 |  | 13 | 4 |  | 12 | 1 | - | 0.355 |
| TAMs= Tumor-associated macrophages; N=Number of patients; TN=Tumor nests; TS=Tumor stroma; IM= invasive margin; primary tumor=PT; KT=Krukenberg tumor. | | | | | | | | | | | | | | | | |
