# Supplementary figures and images for: Invasion characteristics and clinical significance of tumor-associated macrophages in gastrointestinal Krukenberg tumors
Source: Front Oncol. 2023 Feb 24;13:1006183. doi: 10.3389/fonc.2023.1006183 (PMC9999382; doi:10.3389/fonc.2023.1006183)

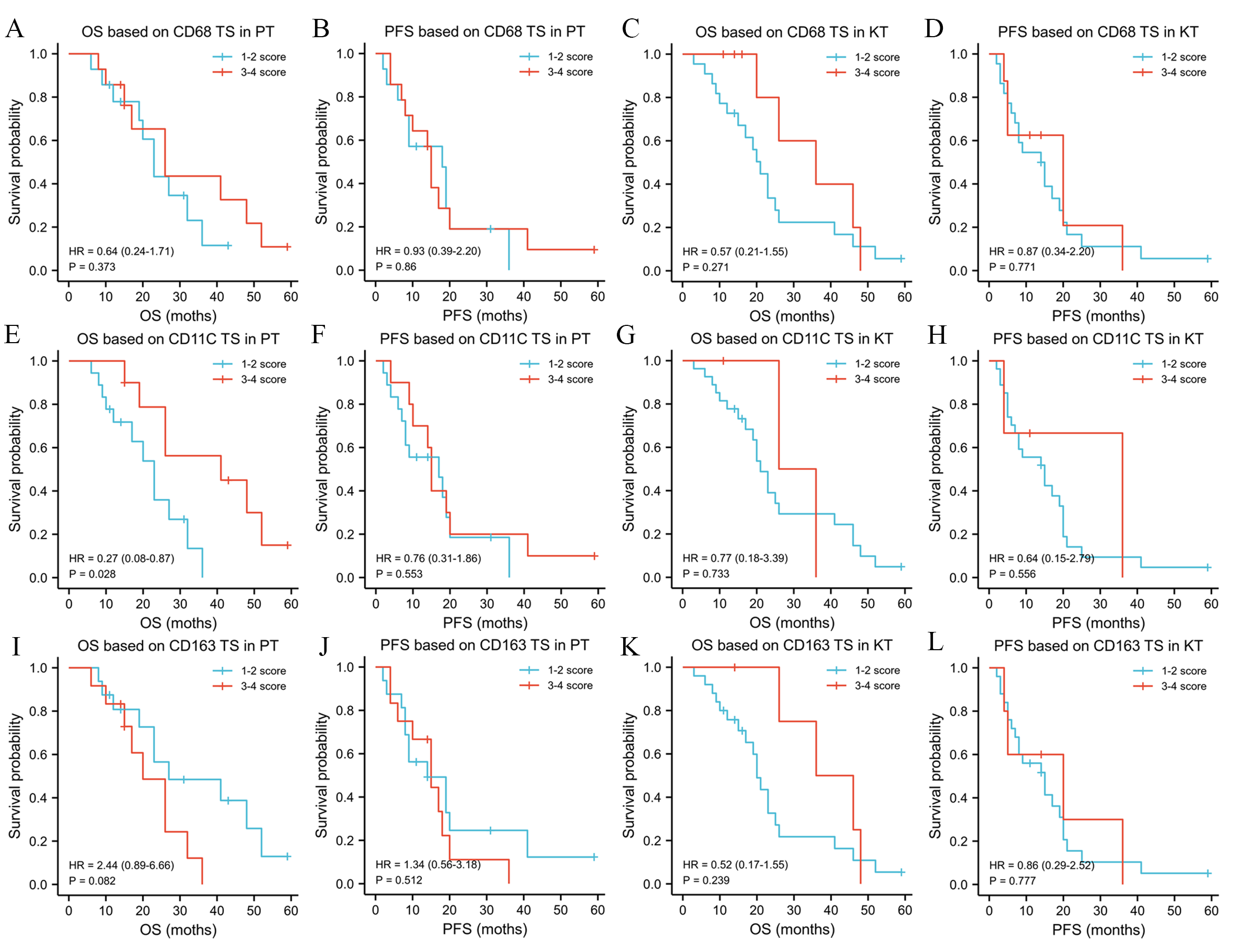

Supplement: Supplementary file 4 [file Image_1.tif]

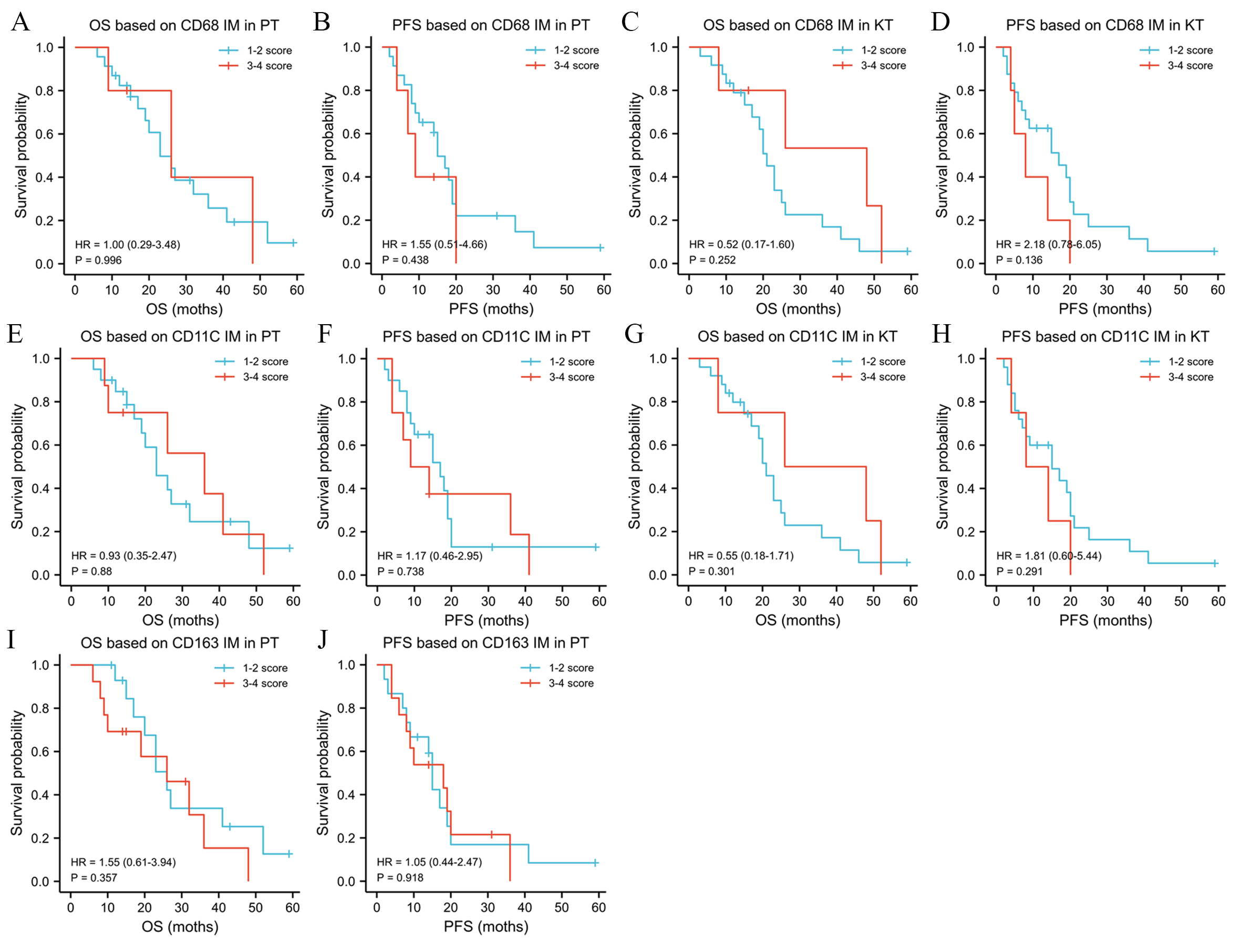

Supplement: Supplementary file 5 [file Image_2.tif]
